# A data-driven model for influenza transmission incorporating media effects

**DOI:** 10.1098/rsos.160481

**Published:** 2016-10-26

**Authors:** Lewis Mitchell, Joshua V. Ross

**Affiliations:** School of Mathematical Sciences, University of Adelaide, North Terrace, 5005 Adelaide, Australia

**Keywords:** epidemiology, influenza, mathematical modelling, social media, Twitter

## Abstract

Numerous studies have attempted to model the effect of mass media on the transmission of diseases such as influenza; however, quantitative data on media engagement has until recently been difficult to obtain. With the recent explosion of ‘big data’ coming from online social media and the like, large volumes of data on a population’s engagement with mass media during an epidemic are becoming available to researchers. In this study, we combine an online dataset comprising millions of shared messages relating to influenza with traditional surveillance data on flu activity to suggest a functional form for the relationship between the two. Using this data, we present a simple deterministic model for influenza dynamics incorporating media effects, and show that such a model helps explain the dynamics of historical influenza outbreaks. Furthermore, through model selection we show that the proposed media function fits historical data better than other media functions proposed in earlier studies.

## 1. Introduction

Traditional models of epidemics assume static parameter values over the course of an outbreak [1]. As such, they do not allow for changes in human behaviour which in turn are likely to impact the rate of transmission in a population. Such behavioural changes in response to disease outbreaks are well established [2]. This includes self-imposed social distancing during influenza pandemics [3], and the usage of face masks and changes in travel behaviour during the severe acute respiratory syndrome (SARS) outbreak of 2002–2004 [4]. The term *prevalence elastic behaviour* has arisen to explain voluntary protective behaviour which increases with disease prevalence [5], as has been observed for both measles [6] and HIV [7].

The close to real-time awareness of disease prevalence in an outbreak is now common due to the relatively recent explosion in mass and social media. The past decade has seen significant growth in studies concerning the interaction of media, human behaviour and infectious disease dynamics, and there now exists a substantial body of work on this topic [2,8–14]. Despite this growth, empirical studies of prevalence elastic behaviour due to mass media have until recently been difficult due to the lack of availability of data directly measuring media engagement and relating it to behavioural change. As such, the vast majority of studies in this area can be broadly classified into two groups, with slightly different motivations. First, *pure mathematical models of behavioural change*, in which a model is formulated that accounts for how dynamics are influenced by disease awareness or prevalence, typically facilitated through media—these are often either in the form of introducing new states which account for the behavioural status of individuals [15], by allowing modification to the contact structure [3,16], or by allowing modification to the model parameters [17,18]—and the consequences are then explored. Collinson *et al.* [13] model behavioural change due to media by explicitly including a compartment for individuals influenced by mass media into an SEIR-type model, also incorporating effects like vaccination and social distancing [13]. This study is of particular interest due to the fact that it incorporates a ‘media fatigue’ effect during the 2009/2010 H1N1 pandemic by fitting to news report data collected from newspaper homepages during the pandemic. Second, *pure statistical models of media and prevalence* are used on large datasets to produce statistical regression models relating some measure of volume of media concerning epidemics to the prevalence of infection [9,10] or reproductive number [19]. Such models have recently become popular due to the rapid increase in new data streams coming from Internet and online social media usage [20,21]. The study of Signorini *et al.* [11] is an exception to this trend: while it is a pure statistical model, it includes an investigation of the relationship between ‘tweets’ on Twitter and public sentiment with respect to H1N1 [11]. The FluOutlook platform [14] is also particularly interesting; by using a variety of data sources, including Twitter, to initialize a global agent-based epidemiological model it is able to produce real-time forecasts of an evolving influenza season.

Here our focus is on simple models for incorporating behavioural changes from awareness of disease prevalence, through modification to the effective transmission rate parameter. We measure disease dynamics through influenza incidence data from the United States over the period 1998/1999–2014/2015, and human behaviour through social media data collected from Twitter over the period 2009/2010–2014/2015. Modification to the effective transmission rate is via a so-called *media function*. Three distinct media functions have been introduced, and recently compared, in the literature [22]. A potential criticism of pure mathematical model-based studies, as described above, is that the usefulness of the model when analysing real data is uncertain. In fact, as we will show here, some of these models have only very limited use in describing data coming from historical influenza outbreaks. On the other hand, while pure statistical models of media and prevalence are potentially of use for detecting and tracking disease incidence, they are subject to typical criticisms of ‘big data’ analyses [23] as containing biases and tending towards overfitting. As such, their usefulness for understanding potential mechanisms of impacts, as is the focus of model-based analyses, is limited.

We propose a data-driven approach that couples these existing paradigms: through a statistical analysis of data on media engagement and disease prevalence we develop a mathematical model of behaviour change which may then be validated against data. Our approach uses online social media data from Twitter alongside surveillance data on influenza to inform the form of the media function. The motivation is that by using both sources of data, we have some empirical justification for the form of the chosen media function and can also better describe real observations. By using model selection criteria, we show that the media function proposed here fits historical surveillance data better than other media functions proposed in earlier studies.

The structure of the remainder of this paper is as follows: in §2, we describe the dataset and model used; in §3, we show results comparing our proposed model with surveillance data, and then conclude with a discussion in §4.

## 2. Material and methods

In order to measure media engagement, we use a corpus of over 2.9 million geolocated, flu-related tweets collected from the contiguous United States between September 2009 and July 2015. This sample was provided by the Computational Story Lab at the University of Vermont, and is a subset of Twitter’s ‘garden hose’ feed, representing approximately 10% of all public messages posted to the platform. In this study, we consider only tweets which contain one or more of the strings ‘flu’, ‘#flu’, ‘influenza’ or ‘#influenza’. Furthermore, we will focus on ‘retweeted’ messages, where an individual has opted to reshare a tweet originally authored by someone else with their own followers by means of a retweet button within the Twitter interface or by appending the string ‘RT’ to the beginning of the original message. Such messages account for approximately 30% of the corpus and are mainly resharings of flu-related articles from major news outlets, but can also contain retweets of messages authored by regular Twitter users.

We use a deterministic SEEIIR-M model (*susceptible–exposed–infected–recovered with media*, with two compartments for exposed and infected individuals) to model the transmission of influenza under the influence of media effects:

2.1 $$\begin{array}{rl} \dot{S} & =-\beta f(I)SI, \end{array}$$

2.2 $$\begin{array}{rl} {\dot{E}}_{1} & =\beta f(I)SI-2\sigma E_{1}, \end{array}$$

2.3 $$\begin{array}{rl} {\dot{E}}_{2} & =2\sigma E_{1}-2\sigma E_{2}, \end{array}$$

2.4 $$\begin{array}{rl} {\dot{I}}_{1} & =2\sigma E_{2}-2\gamma I_{1}, \end{array}$$

2.5 $$\begin{array}{rl} {\dot{I}}_{2} & =2\gamma I_{1}-2\gamma I_{2} \end{array}$$

2.6 $$\begin{array}{rl} \;\mathrm{and}\;\dot{R} & =2\gamma I_{2}, \end{array}$$

where *S*,*E*_1_,*E*_2_,*I*_1_,*I*_2_ and *R* represent the proportions of the population in each compartment, *S*+*E*_1_+*E*_2_+*I*_1_+*I*_2_+*R*=1, *β* represents the effective transmission rate in the absence of media effects, 1/*σ* represents the average latent period, 1/*γ* represents the average infectious period and *f*(*I*) is the so-called *media function* which represents the reduction in transmission of the disease through the influence of mass media. Consequently, 0≤*f*(*I*)≤1 with *f*(*I*)≡1 implying no effect of media upon transmission, and we will assume *f*(*I*) is monotonically decreasing in *I*. Setting *f*(*I*)≡1 recovers the standard SEEIIR model. As *f*(0)=1 for each media function, the basic reproduction number *R*_0_=*β*/*γ*, which is independent of *f*(*I*). The two compartments for the exposed and infectious periods mean that these periods have underlying Erlang-2 distributions with mean exposed and infectious periods 1/*σ* and 1/*γ* respectively, which have been shown to more accurately represent the shape of observed distributions [24]. We found similar results using standard SEIR-type models; these results are presented in the electronic supplementary material. Note that we have not included vaccination in our model, for two reasons: firstly, for comparison with media models from previous studies (see below) which use SEIR-type models without vaccination; and secondly, because vaccination coverage in adults has remained approximately constant since 2010 [25] and the Twitter data we will study primarily relates to media reporting around the peak of the influenza season rather than the earlier peak of the vaccination season. Our model, therefore, can essentially be considered as a model for influenza dynamics in the unvaccinated portion of the population.

Previous studies have postulated a number of different forms for *f*(*I*); see [22] for a recent review. In particular, Cui *et al.* [26] set

2.7 $$f_{1}(I)=\exp(-p_{1}I)$$

within an SEI model, Xiao & Ruan [27] used

2.8 $$f_{2}(I)=\frac{1}{1+p_{2}I^{2}}$$

within an SIR model to account for the psychological effects of a large population infected with SARS, and many authors (e.g. [28,29]) set

2.9 $$f_{3}(I)=\frac{1}{1+p_{3}I}$$

to account for various effects including media coverage.

To compare the model outputs with real data, we use influenza surveillance data provided by the US Centre for Disease Control (CDC) [25]. Specifically, we fit models to the nation-wide percentage of new laboratory-confirmed influenza cases per week. We find best fits for the free model parameters to the surveillance data by minimizing least-square error between model solutions and surveillance data using a limited memory Broyden–Fletcher–Goldfarb–Shanno (L-BFGS-M) method [30], implemented in Python. To ensure the numerical stability of the numerical optimization routine, we constrain *R*_0_ to be between 1 and 2, the mean infectious period 1/*γ* to be between 1 and 5 days, and the mean exposed time 1/*σ* to be between 1 and 3 days. To perform model selection, we use the Akaike Information Criterion (AIC) with finite sample size correction.

## 3. Results

**Figure 1.**
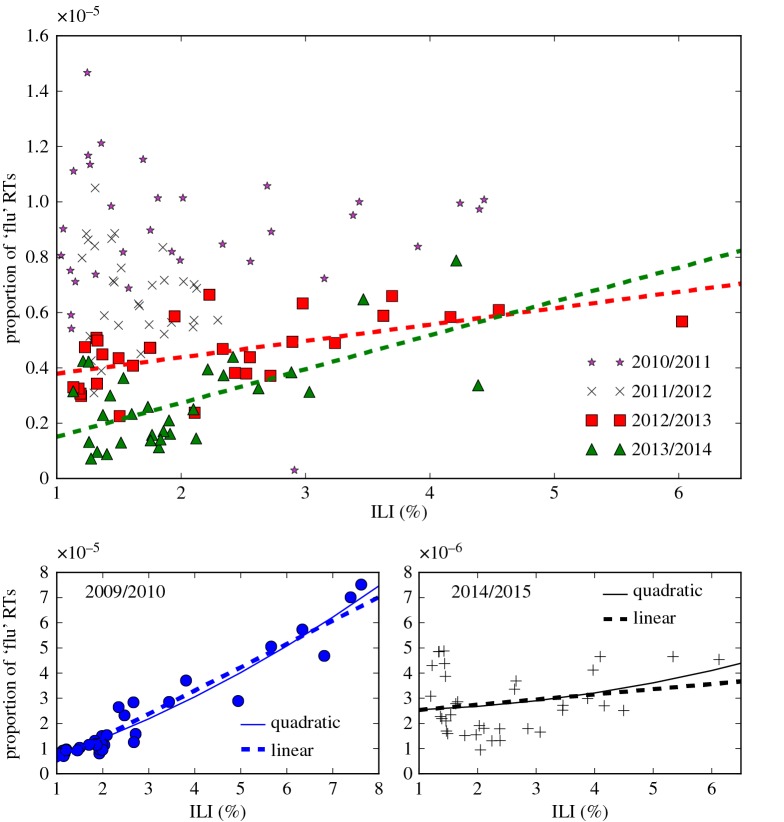
Media engagement from Twitter data. Correlation between proportion of public retweets regarding ‘flu’ and number of influenza-like-illness (ILI) cases, 2009/10–2014/15. ILI data are expressed as a percentage of the total number of visits to sentinel surveillance providers. Linear trend lines are shown for the years showing significant (*p*<0.01) correlation. The subfigures show both quadratic and linear fits to the data for the 2009/2010 and 2014/2015 seasons.

We use the act of sharing a message pertaining to influenza as a proxy for an individual engaging with media about an influenza outbreak. While this act of sharing does not necessarily imply that the individual will change their behaviour, it does suggest that the individual is at least somewhat *concerned* by the media surrounding the influenza outbreak. Figure 1 shows the relationship between proportion of US-based tweets which were retweets concerning influenza (that is, number of retweets containing one or more of the strings ‘influenza’, ‘flu’, ‘#influenza’ or ‘#flu’ divided by the total number of tweets) and the number of influenza-like-illness (ILI) cases per week for the 2009/2010 to 2014/2015 influenza seasons, expressed as a percentage of the total number of visits to sentinel providers. The data on weekly counts of ILI activity and retweeting rates used can be found in the electronic supplementary material. We chose to fit to ILI activity rather than laboratory-confirmed influenza incidence because we expect individuals to tend to share flu-related information on social media upon feeling ill, rather than strictly once they are confirmed to have influenza. The 2009/2010 pandemic (plotted in the lower left subplot) stands out as having the largest number of both ILI cases and retweet activity. We observe strong Pearson’s correlations between retweets and influenza activity for 3 out of the 6 years plotted—in 2009/2010, 2012/2013 and 2013/2014 (*p*<0.01).

Importantly, while the relationship between media engagement and flu activity is small, it is roughly linear for most flu seasons plotted. Using AIC to test linear and quadratic models for the data, we found that the linear model was selected in all seasons apart from 2009/2010. We show linear and quadratic fits for this season as well as 2014/2015 in the subplots below the main figure. In 2014/2015, the linear model was slightly preferred with a relative Akaike weight of 0.58 to 0.42 for the quadratic model, while in 2009/2010 the quadratic model was slightly preferred with a relative Akaike weight of 0.55 to 0.45 for the linear model. Note that as demonstrated by the model fits in the two subfigures, the Akaike weights indicate that there is substantial support in the data for both the linear and quadratic models. Indeed, we found that the relative likelihood of the quadratic model increased with the total number of ILI cases per season (see electronic supplementary material), suggesting that nonlinear media effects may become increasingly relevant during more severe outbreaks. We also present residual plots for the linear and quadratic models for all years in the electronic supplementary material, showing no obvious non-random patterns for the model fits, along with further details of the AIC model selection and a table of relative Akaike weights for all years. Note also that we observed similar-looking relationships between media engagement and influenza activity when using the number of comments on flu-related articles in the *New York Times* between 2001 and 2013 as our metric for media engagement. However, due to the smaller amount of data we could only find a statistically significant correlation between the two during the 2009 pandemic.

Based upon these observations and for simplicity in comparing models, we propose the following simple linear media function to describe the reduction in transmission due to media effects:

3.1 $$f_{m}(I)=1-p_{m}I,$$

where *p*_*m*_ is a parameter (to be fitted) describing the reduction in actual transmission due to concern from media coverage. Yorke & London applied a similar function in a different context, to model exposure rates for seasonal measles outbreaks [31]. Note that in order to assure that 0≤*f*_*m*_(*I*)≤1 it will be necessary to constrain *p*_*m*_ such that 0≤*p*_*m*_≤1, as *I*≤1 always. This is in contrast with the media functions (2.7)–(2.9), for which the parameters can take on any value *p*_1_,*p*_2_,*p*_3_≥0. We remark that while an obvious extension for larger outbreaks would be to use a quadratic media function *f*_*m*_(*I*)=1−*p*_*m*1_*I*−*p*_*m*2_*I*^2^, for ease of comparison with existing media functions, we will only consider the one-parameter model (3.1).

**Figure 2.**
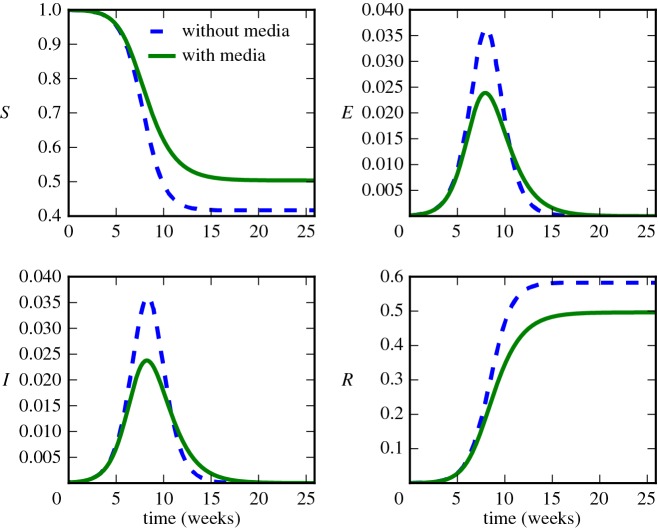
Sample time series showing the effect of the media function *f*_*m*_ (*I*)=1−*p*_*m*_*I*. The media function reduces the final epidemic size and slows the decay rate of the outbreak.

We show an example of the effect of the media function *f*_*m*_ upon the dynamics in figure 2, where we have set *p*_*m*_=0.05, *R*_0_=1.5, *γ*=1/2 (d)^−1^, *σ*=1/2 (d)^−1^ and have plotted *E*=*E*_1_+*E*_2_ and *I*=*I*_1_+*I*_2_. The media function reduces the total number of infected persons (i.e. the final size of the epidemic) and size of the peak, while not notably changing the timing of the peak. The slower rate of depletion of susceptibles means that the infection dies out slightly slower in the model with media effect.

To investigate how well the various transmission models, both with and without media effects, describe real influenza outbreaks, we fit (2.1)–(2.6) with *f*(*I*)≡1 as well as (2.7)–(3.1) to weekly laboratory-confirmed influenza incidence data for the 1998–2013 flu seasons using least squares. Note that unlike social media engagement which can be reasonably expected to relate to ILI, it is appropriate to fit models of the underlying disease dynamics to confirmed influenza incidence data only. Using the L-BFGS-B method, we find parameter values *R*_0_, *σ*, *γ* and media parameter *p*_*m*_ which best fit the data. The best-fitting parameters for each model for the 2013/2014 flu season are shown in table 1, and for all other seasons are shown in the electronic supplementary material. We fit observations from four weeks before the peak to 12 weeks after the peak. Also shown in table 1 are the average conditional probabilities for each model, as obtained from the normalized Akaike weights for each model across all flu seasons between 1998/1999 and 2014/2015 in which a non-zero media function was found.

**Table 1.** Parameters of best fit for SEEIIR and SEEIIR-M models for 2013/2014 influenza season.

	*f*(*I*)≡1	*f*_*m*_	*f*_1_	*f*_2_	*f*_3_
*R*_0_	1.1574	1.5101	1.8574	1.4949	1.9281
1/*σ* (days)	1	1.6881	2.2162	1.9873	1.3794
1/*γ* (days)	1.1979	1	1.2719	1.0979	1.1001
*p*_*i*_	—	0.3316	0.1543	0.7381	0.8140
*p*_AIC_	*O* (10^−9^)	>0.9999	*O* (10^−8^)	*O* (10^−7^)	*O* (10^−5^)

**Figure 3.**
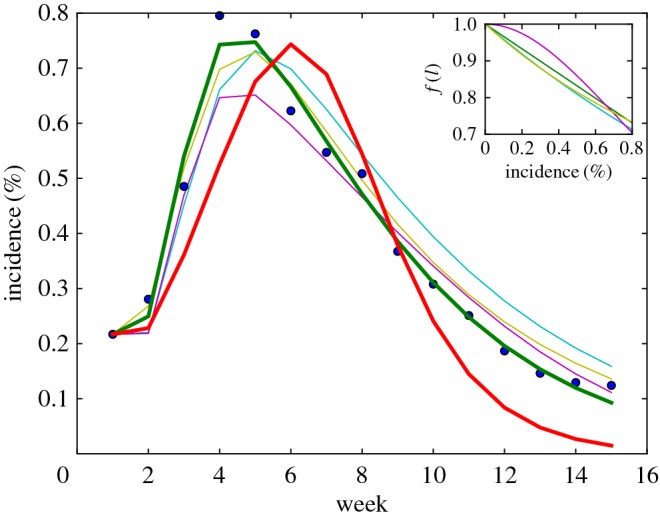
Best model fits to data from 2013/2014 flu season. Weekly laboratory-confirmed influenza incidence data (blue dots), and model fits for SEEIIR model without media function (red), with media function (green) and variations *f*_1_ (cyan), *f*_2_ (magenta) and *f*_3_ (yellow), for the 2013/2014 influenza season (USA). Incidence data are normalized by the total number of visits to sentinel providers.

In figure 3, we show example fits to observations of the percentage of new laboratory-confirmed influenza cases per week (blue) for the model with no media effect (red) and media functions given by *f*_*m*_ (green), *f*_1_ (cyan), *f*_2_ (magenta) and *f*_3_ (yellow) for the 2013/2014 influenza season. As with the ILI data, the laboratory-confirmed case data are expressed as a percentage of the total number of visits to sentinel surveillance providers. The inset plot shows the corresponding media functions with best-fitting parameters. While no model is able to correctly estimate the size of the peak of the infection, the model with linear media function *f*_*m*_ is the only one which correctly identifies the week in which the infection peaks. The media functions *f*_*m*_ and *f*_1_ are also the only models which describe the decay of the infection post-peak well.

While the media function *f*_*m*_(*I*) was derived based upon Twitter data, our intention in focusing on news-sharing behaviours is to model the effects of mass media more generally. Indeed, we might expect that population-level engagement with other forms of mass media show a similar monotonically decreasing relationship between media coverage and transmission. To that end, we now apply the proposed media function to all influenza seasons we have incidence data for, and find similar results for most seasons between 1998/1999 and 2014/2015. Table 2 shows the average conditional probability of selecting each model, where the average is taken over all years in which a media function is required at all. Also shown are the 95% confidence intervals for each average conditional probability. No media functions of any kind were required to describe the 2003/2004 flu season, *f*_2_ gave the best fit to observations in 2006/2007 only, and *f*_1_ gave the best fit in 2009/2010 only.

**Table 2.** Average probability of selecting each model over the 1998/1999–2014/2015 seasons. We have fitted over a 16-week period for each season. The 2009/2010 pandemic influenza season has been excluded.

	*p*_AIC_	95% CI
*f* (*I*)≡1	0.0500	[0,0.1398]
*f*_*m*_ (*I*)=1−*p*_*m*_*I*	0.8347	[0.6674,1]
$f_{1}\;(I)=\exp(-\gamma p_{1}I)$	0.0267	[0,0.0535]
$f_{2}\;(I)=\frac{1}{1+p_{2}I^{2}}$	0.0060	[0.0001,0.0120]
$f_{3}\;(I)=\frac{1}{1+p_{3}I}$	0.0826	[0,0.1871]

**Figure 4.**
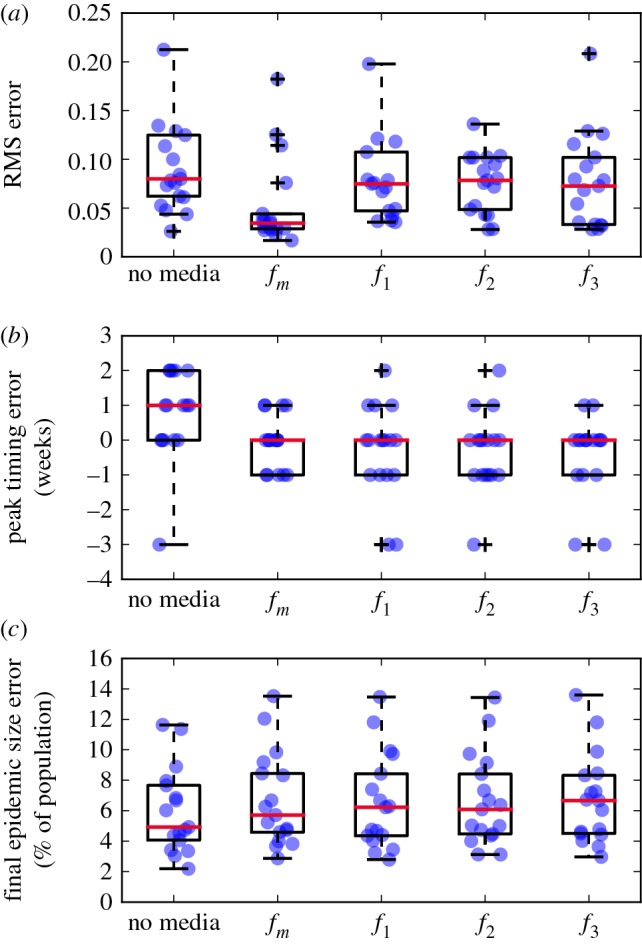
Boxplots of RMS error (*a*), peak timing error (*b*) and final epidemic size error (*c*), for standard model and models with media effects for 1998/1999–2014/2015 seasons. The 2009/2010 pandemic influenza season has been excluded. Blue dots show results from individual years (where we have added random jitter for visibility), crosses show outliers.

We next examine how well models with and without media function estimate the complete epidemic curve, as well as the peak timing and severity. Figure 4 shows boxplots of (i) RMS error, (ii) peak timing error and (iii) final epidemic size error, for the model with no media effect as well as media functions as defined in (2.7)–(3.1) over the 1998/1999–2014/2015 seasons. The proposed media function *f*_*m*_ significantly outperforms all other models with or without media effects at fitting the epidemic curve, with the distribution of RMS errors significantly less spread and centred closer to 0 than all other models. All models with media effect are significantly better than the standard model at matching the observed peak timing of an outbreak (Mood’s median test, *p*=0.05), although there is no significant difference between the four models. Similarly, there is no significant difference across models in explaining the observed final epidemic size, in fact the median error for the standard model without media effect is slightly lower than that for the models with media functions (however, this difference is not significant).

**Figure 5.**
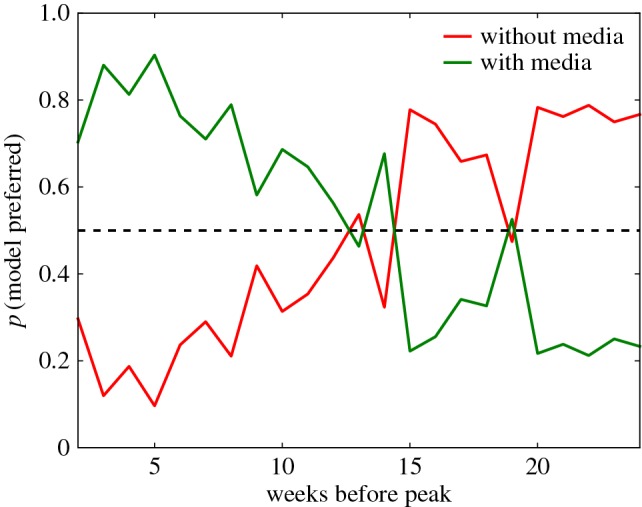
Quality of model fits as a function of lead time. Probability that the no-media model (green) and media model with *f*_*m*_(*I*)=1−*p*_*m*_*I* (blue) are the better model as a function of number of weeks before peak. In each case, we fit models to data over a 16-week period. The 2009/2010 pandemic influenza season has been excluded.

We remark that much of the improvement made by the media function *f*_*m*_ comes from better describing the post-peak period. The no media (i.e. *f*(*I*)≡1) model becomes preferable as more of the data leading up to the peak of each season is used to fit the models. In figure 5, we show the average conditional probability of selecting each model as a function of the number of weeks of data used before the peak. The no media and *f*_*m*_ models are always preferred over the other media function models (i.e. using *f*_1_, *f*_2_ and *f*_3_), with the *f*_*m*_ model being preferred up until around 10–12 weeks before the peak. When fitting data earlier than 12 weeks before the peak the no media model is preferred, suggesting that the effect of media coverage becomes more important later in the season. Furthermore, neither model is able to reliably predict the peak of the infection in terms of either size or timing based upon data from before the peak only. This suggests that in order to make accurate predictions and estimate parameters rather than explain an existing dataset when only small amounts of data are available, we must use a more advanced methodology such as data assimilation [32].

## 4. Discussion

Mass media is clearly an important tool for changing peoples’ behaviour during disease outbreaks. A better understanding of the relationship between media coverage of outbreaks and subsequent behavioural change can aid mathematical modelling efforts, as well as the development of public policy around the best use of this resource to inform the public and control the spread of a disease.

By using data collected from Twitter, we have proposed a new, simple media function to describe the reduction in disease transmission due to media effects. When incorporated into a deterministic SEEIIR model, this media function describes incidence data better than a model without media effects, and better than previously proposed media functions. We observed a relationship between outbreak size and media awareness, with a quadratic model becoming more likely as the final size of the outbreak increased. This suggests that the relationship between media coverage and infection rates is nonlinear, especially in more severe seasons. Future extensions to the media function could incorporate extra reductions in disease transmission due to factors such as early media coverage, pre-existing immunity or seasonality. Public awareness campaigns could lead to an increase in early-season social media activity and sharing of news articles, and could be implemented in the current model via a time lag. Indeed, we observed such an effect for the 2014/2015 season, where changes in retweeting activity preceded ILI rates by a number of weeks. Mass media campaigns have been shown to increase flu-related hospital visits [33] and vaccination rates [34,35]. It is further possible that any potential reduction in transmission in one season due to the effects of mass media could decrease pre-existing immunity for the next season, an effect which could be modelled by conditioning the media function on the total amount of media engagement from the previous season. Identifying any such potential process is of course confounded by the presence of multiple influenza strains circulating in any particular season with differing levels of pre-exisitng immunity; modelling such a hierarchy of time-lagged effects requires a more sophisticated strategy and is left for future work.

The interplay between mass media, social network influence, human behavioural change and disease transmission is complex, and this work merely scratches the surface of the processes which could be modelled using this framework. Further extensions could build upon efforts to incorporate interactions between social and contact network structures into the model [36] by inferring the mass media effect directly from social network data. There is also an emerging body of work around using open data to infer human behaviours such as mobility patterns [37] and voluntary avoidance [38]. The same data used here to track media engagement could potentially be exploited to quantify such effects, as well as to develop a proxy for real-time surveillance on practices such as vaccination, which we aim to incorporate into future refinements of this model.

A critical assumption made in this work is that the population is homogeneously mixing and not age-stratified. This is of course far from being the case for Twitter users—indeed, it is well known that the demographics of Twitter use in the United States are biased towards adults aged 18–29, African-Americans and urban residents [39], and word usage has been shown to correlate with a number of socio-economic and health characteristics [40,41]. Despite these biases, the approximately 10% of American adults who are estimated to use Twitter represents a far larger sample size than those of traditional surveys. Furthermore, for simplicity and because the keywords we used were sufficiently specific, we did not filter tweets for relevance. Manual examination of a sample of tweets indicated that an insignificant number of tweets were misclassified as being about influenza; however, constraining the tweet corpus may lead to further improvements in the results.

This work fits into a growing field of research on disease prediction using open data [42], particularly from social network usage. Great advances have already been made on algorithms to predict rare and seasonal diseases, especially in the computer science literature [43]. Our results represent a first attempt at incorporating this emerging data stream into more traditional modelling efforts, and hopefully at better understanding the interactions between media and disease dynamics.

## Supplementary Material

appendix.pdf: Appendix containing extra information regarding media function model selection from Twitter data. - fluTwitterData.csv: Data file containing weekly ILI and tweet counts.

## Data accessibility

Data available from the Dryad Digital Repository at http://dx.doi.org/10.5061/dryad.593cc [44].
