# Supplementary material for: A data-driven model for influenza transmission incorporating media effects
Source: R Soc Open Sci. 2016 Oct 26;3(10):160481. doi: 10.1098/rsos.160481 (PMC5098988; doi:10.1098/rsos.160481)
Supplement: appendix.pdf: Appendix containing extra information regarding media function model selection from Twitter data. - fluTwitterData.csv: Data file containing weekly ILI and tweet counts. [file rsos160481supp1.pdf]

# A data-driven model for influenza transmission incorporating media effects

Lewis Mitchell & Joshua V. Ross

## Appendix

In Figure 1 we show the residuals from linear (blue circles) and quadratic (red crosses) least-square fits to the retweet-infected data from Figure 1 of the main text, for all seasons 2009/10-2014/15 considered in this study. Table 1 shows the relative Akaike weights for the linear and quadratic models for each year, where the relative weights $p_i$ are given by

$$p_i = \exp\left(\frac{AIC_{\min} - AIC_i}{2}\right)$$

and $AIC_i$ are the AIC values for each model, $AIC_{\min}$ is the minimum value of $AIC_i$. These weights represent the probability that the $i$th model minimises the information loss in describing the data, relative to the best-fitting model [1]. Figure 2 shows relative Akaike weight of the quadratic model as a function of final outbreak size, suggesting that nonlinear effects will increasingly come into play for more severe outbreaks and pandemics.

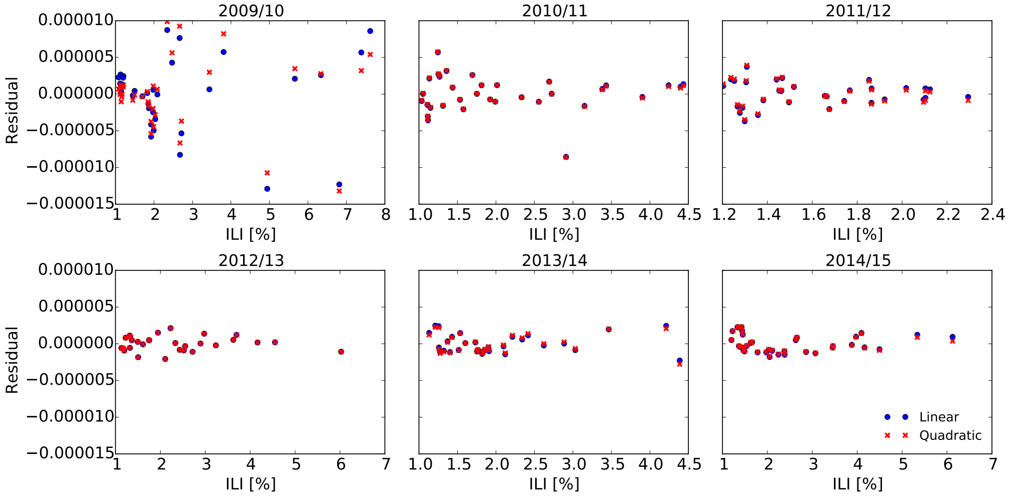


**Figure 1.** Residual plots for linear (blue dots) and quadratic (red crosses) media function models.

**Table 1.** Relative Akaike weights $p_{\text{lin}}$ and $p_{\text{quad}}$ for selecting the linear and quadratic models during each influenza season 1998/99-2014/15. Weights for each preferred model are shown in bold.

| year | $p_{\text{lin}}$ | $p_{\text{quad}}$ |
|---|---|---|
| 2009/10 | 0.4454 | **0.5546** |
| 2010/11 | **0.7769** | 0.2231 |
| 2011/12 | **0.8211** | 0.1789 |
| 2012/13 | **0.7781** | 0.2219 |
| 2013/14 | **0.6966** | 0.3034 |
| 2014/15 | **0.5811** | 0.4189 |

rsos.royalsocietypublishing.org R. Soc. open sci. 000000

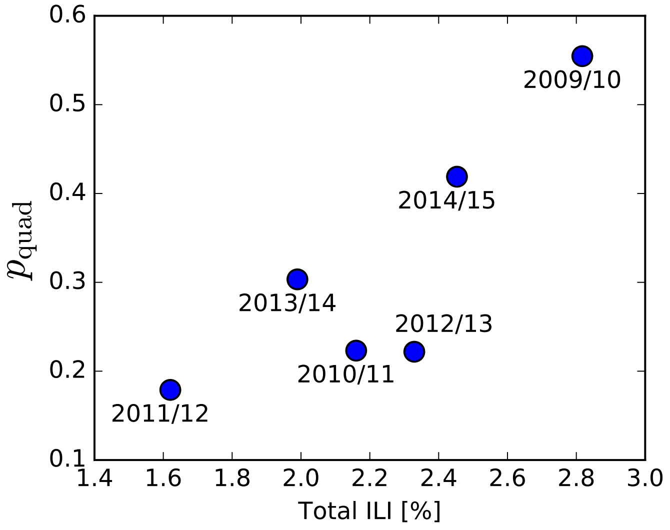


**Figure 2.** Relationship between relative Akaike weight for the quadratic model $p_{\text{quad}}$ and the observed final outbreak size for each influenza season 2009/10-2014/15.

Table 2 shows the best-fitting parameters for the SEEIIR and SEEIIR-M models for all seasons 1998/99-2014/15, analogously to Table 1 in the main text. For completeness we also present results where we have used a standard SEIR model with only one exposed and infectious compartment each. Table 3 is an analog of Table 2 from the main text, showing the average conditional probability of selecting each model, and Figure 3 shows example fits to observations of the percentage of new laboratory-confirmed influenza cases per week for the SEIR and SEEIIR-M models, similarly to Figure 3 in the main text. In terms of model fitting, the confidence intervals in Table 3 show no significant difference in the likelihood of selecting each media model. Figure 3 suggests that in 2013/14 the media functions $f_1$, $f_2$ and $f_3$ fit the data slightly worse using an SEIR model than with an SEEIIR model, while the $f(I) \equiv 1$ and $f_m$ fit describe the data essentially as well using an SEIR model than with an SEEIIR model.

Table 2: Parameters of best fit for SEEIIR and SEEIIR-M models for all influenza seasons.

| | model | $R_0$ | $1/\sigma$ (days) | $1/\gamma$ (days) | $p_i$ | $p_{AIC}$ |
|---|---|---|---|---|---|---|
| 1998 | $f(I) \equiv 1$ | 1.17 | 1.00 | 2.41 | – | 0.80 |
| | $f_m$ | 2.00 | 1.92 | 1.41 | 0.22 | 0.02 |
| | $f_1$ | 1.30 | 1.00 | 2.30 | 0.02 | 0.15 |
| | $f_2$ | 1.35 | 1.21 | 2.06 | 0.06 | 0.03 |
| | $f_3$ | 1.58 | 1.98 | 2.02 | 5.71 | 0.00 |
| 1999 | $f(I) \equiv 1$ | 1.23 | 1.00 | 1.63 | – | 0.00 |
| | $f_m$ | 1.47 | 1.28 | 1.58 | 0.11 | 0.99 |
| | $f_1$ | 1.63 | 1.71 | 1.38 | 0.03 | 0.01 |
| | $f_2$ | 2.00 | 1.95 | 1.92 | 0.62 | 0.00 |
| | $f_3$ | 1.76 | 1.90 | 1.30 | 4.14 | 0.01 |
| | $f(I) \equiv 1$ | 1.14 | 1.00 | 1.26 | – | 0.00 |
| | $f_m$ | 1.30 | 1.00 | 1.23 | 0.21 | 1.00 |

Continued on next page

| | model | $R_0$ | $1/\sigma$ (days) | $1/\gamma$ (days) | $p_i$ | $p_{AIC}$ |
|---|---|---|---|---|---|---|
| 2000 | $f_1$ | 1.35 | 1.13 | 1.13 | 0.04 | 0.00 |
| | $f_2$ | 1.33 | 1.99 | 1.97 | 1.05 | 0.00 |
| | $f_3$ | 1.59 | 1.99 | 1.99 | 1.06 | 0.00 |
| | $f(I)\equiv 1$ | 1.10 | 1.00 | 1.98 | – | 0.00 |
| | $f_m$ | 1.45 | 1.14 | 1.33 | 0.24 | 0.89 |
| 2001 | $f_1$ | 1.34 | 1.37 | 1.32 | 0.03 | 0.00 |
| | $f_2$ | 1.38 | 1.29 | 1.37 | 0.26 | 0.00 |
| | $f_3$ | 1.46 | 1.07 | 1.38 | 3.15 | 0.11 |
| | $f(I)\equiv 1$ | 1.13 | 1.00 | 1.00 | – | 0.00 |
| | $f_m$ | 1.28 | 1.00 | 1.00 | 0.29 | 1.00 |
| 2002 | $f_1$ | 1.56 | 1.61 | 1.55 | 0.19 | 0.00 |
| | $f_2$ | 1.28 | 1.35 | 1.00 | 0.70 | 0.00 |
| | $f_3$ | 1.71 | 1.83 | 1.74 | 0.54 | 0.00 |
| | $f(I)\equiv 1$ | 1.17 | 1.00 | 1.46 | – | 1.00 |
| | $f_m$ | 1.17 | 1.00 | 1.46 | 0.00 | 0.00 |
| 2003 | $f_1$ | 1.17 | 1.00 | 1.46 | 0.00 | 0.00 |
| | $f_2$ | 1.17 | 1.00 | 1.46 | 0.00 | 0.00 |
| | $f_3$ | 1.19 | 1.00 | 1.46 | 56.78 | 0.00 |
| | $f(I)\equiv 1$ | 1.11 | 1.00 | 1.30 | – | 0.00 |
| | $f_m$ | 1.36 | 1.02 | 1.00 | 0.30 | 1.00 |
| 2004 | $f_1$ | 2.00 | 2.00 | 2.07 | 0.68 | 0.00 |
| | $f_2$ | 1.23 | 1.16 | 1.10 | 0.41 | 0.00 |
| | $f_3$ | 1.14 | 1.09 | 1.20 | 33.94 | 0.00 |
| | $f(I)\equiv 1$ | 1.10 | 1.00 | 1.09 | – | 0.00 |
| | $f_m$ | 1.24 | 1.00 | 1.01 | 0.28 | 1.00 |
| 2005 | $f_1$ | 1.10 | 1.78 | 2.95 | 0.14 | 0.00 |
| | $f_2$ | 1.33 | 1.00 | 1.00 | 2.58 | 0.00 |
| | $f_3$ | 1.19 | 1.00 | 1.29 | 4.23 | 0.00 |
| | $f(I)\equiv 1$ | 1.12 | 1.00 | 1.22 | – | 0.00 |
| | $f_m$ | 1.40 | 1.00 | 1.00 | 0.36 | 1.00 |
| 2006 | $f_1$ | 1.32 | 1.04 | 1.16 | 0.05 | 0.00 |
| | $f_2$ | 1.62 | 1.04 | 1.00 | 2.51 | 0.00 |
| | $f_3$ | 1.54 | 1.78 | 1.00 | 1.24 | 0.00 |
| | $f(I)\equiv 1$ | 1.12 | 1.00 | 1.64 | – | 0.01 |
| | $f_m$ | 1.26 | 1.00 | 1.42 | 0.13 | 0.99 |
| 2007 | $f_1$ | 1.45 | 1.49 | 1.09 | 0.04 | 0.00 |
| | $f_2$ | 1.72 | 1.27 | 1.00 | 0.66 | 0.00 |
| | $f_3$ | 1.42 | 1.84 | 1.84 | 2.11 | 0.00 |
| | $f(I)\equiv 1$ | 1.14 | 2.83 | 1.06 | – | 0.00 |
| | $f_m$ | 1.28 | 1.00 | 1.07 | 0.27 | 0.96 |
| 2008 | $f_1$ | 1.40 | 1.13 | 1.00 | 0.06 | 0.00 |
| | $f_2$ | 1.25 | 1.02 | 1.02 | 0.57 | 0.04 |
| | $f_3$ | 2.00 | 1.18 | 1.13 | 0.48 | 0.00 |
| | $f(I)\equiv 1$ | 1.16 | 1.00 | 1.74 | – | 0.00 |
| | $f_m$ | 1.10 | 1.10 | 3.39 | 0.06 | 0.00 |
| 2009 | $f_1$ | 1.39 | 1.28 | 1.46 | 0.03 | 0.91 |
| | $f_2$ | 1.41 | 1.64 | 1.19 | 0.11 | 0.00 |
| | $f_3$ | 1.53 | 1.57 | 1.28 | 4.83 | 0.09 |
| | $f(I)\equiv 1$ | 1.11 | 1.00 | 1.31 | – | 0.35 |

Continued on next page

| | model | $R_0$ | $1/\sigma$ (days) | $1/\gamma$ (days) | $p_i$ | $p_{AIC}$ |
|---|---|---|---|---|---|---|
| 2010 | $f_m$ | 1.16 | 1.28 | 1.04 | 0.06 | 0.00 |
| | $f_1$ | 1.30 | 1.86 | 2.32 | 0.22 | 0.00 |
| | $f_2$ | 1.28 | 1.02 | 1.01 | 0.33 | 0.56 |
| | $f_3$ | 1.21 | 1.00 | 1.16 | 7.44 | 0.09 |
| 2011 | $f(I)\equiv 1$ | 1.10 | 2.75 | 1.09 | – | 0.00 |
| | $f_m$ | 1.24 | 1.00 | 1.00 | 0.48 | 1.00 |
| | $f_1$ | 1.57 | 1.00 | 1.33 | 0.38 | 0.00 |
| | $f_2$ | 1.10 | 1.03 | 1.42 | 0.35 | 0.00 |
| | $f_3$ | 1.25 | 1.09 | 1.56 | 1.24 | 0.00 |
| 2012 | $f(I)\equiv 1$ | 1.15 | 1.00 | 1.94 | – | 0.00 |
| | $f_m$ | 1.44 | 1.30 | 1.70 | 0.21 | 0.96 |
| | $f_1$ | 1.57 | 1.63 | 1.51 | 0.07 | 0.01 |
| | $f_2$ | 1.41 | 1.51 | 1.57 | 0.22 | 0.00 |
| | $f_3$ | 1.54 | 1.44 | 1.61 | 2.96 | 0.03 |
| 2013 | $f(I)\equiv 1$ | 1.16 | 1.00 | 1.25 | – | 0.00 |
| | $f_m$ | 1.53 | 1.78 | 1.00 | 0.34 | 1.00 |
| | $f_1$ | 1.68 | 2.13 | 1.01 | 0.07 | 0.00 |
| | $f_2$ | 1.33 | 1.33 | 1.19 | 0.35 | 0.00 |
| | $f_3$ | 1.75 | 1.23 | 1.13 | 1.19 | 0.00 |
| 2014 | $f(I)\equiv 1$ | 1.19 | 1.00 | 2.46 | – | 0.00 |
| | $f_m$ | 1.70 | 1.67 | 2.00 | 0.18 | 0.81 |
| | $f_1$ | 1.65 | 2.00 | 2.02 | 0.06 | 0.00 |
| | $f_2$ | 1.57 | 1.68 | 2.02 | 0.12 | 0.02 |
| | $f_3$ | 1.96 | 1.93 | 1.85 | 2.64 | 0.17 |

**Table 3.** Average probability of selecting each SEIR-type model over the 1998/99-2014/15 seasons. (cf. Table 2 of the main text.) The 2009/10 pandemic influenza season has been excluded.

| | $p_{AIC}$ | 95% CI |
|---|---|---|
| $f(I)\equiv 1$ | 0.0635 | [0,0.1714] |
| $f_m(I)=1-p_mI$ | 0.9122 | [0.7974,1] |
| $f_1(I)=\exp(-\gamma p_1 I)$ | 0.0010 | [0,0.0026] |
| $f_2(I)=\frac{1}{1+p_2I^2}$ | 0.0134 | [0,0.0388] |
| $f_3(I)=\frac{1}{1+p_3I}$ | 0.0100 | [0,0.0255] |

# References

1 Anderson DR. Model Based Inference in the Life Sciences. New York: Springer; 2008.

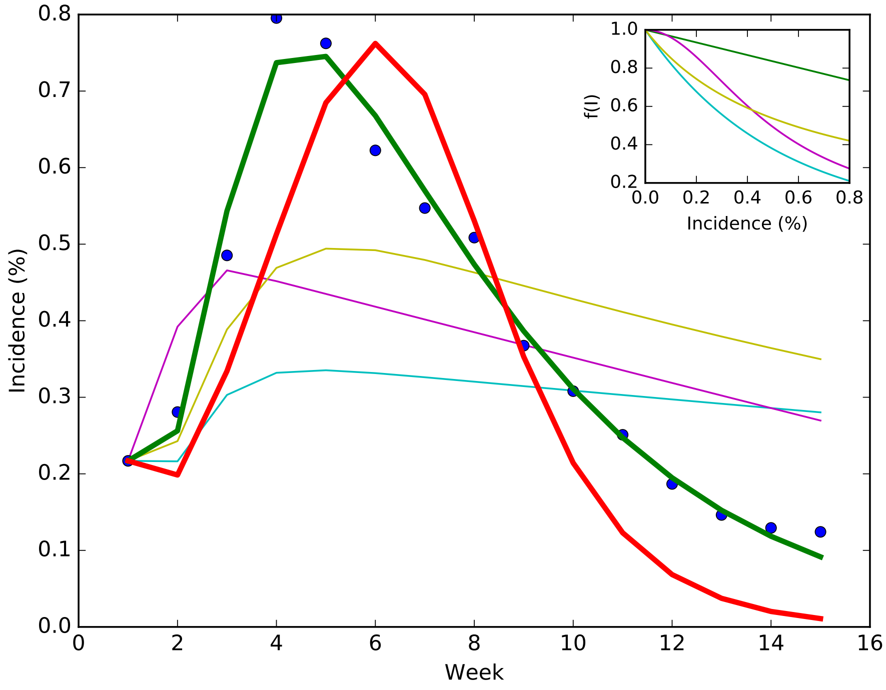


**Figure 3.** Best model fits to data from 2013/14 flu season. Weekly influenza data (blue dots), and model fits for SEIR model without media function (red), with media function (green), and variations $f_1$ (cyan), $f_2$ (magenta) and $f_3$ (yellow), for the 2013/14 influenza season (USA). (cf. Figure 3 from the main text.)
